# Prenylated Phenol and Benzofuran Derivatives from *Aspergillus terreus* EN-539, an Endophytic Fungus Derived from Marine Red Alga *Laurencia okamurai*

**DOI:** 10.3390/md17110605

**Published:** 2019-10-24

**Authors:** Hong-Lei Li, Xiao-Ming Li, Sui-Qun Yang, Ling-Hong Meng, Xin Li, Bin-Gui Wang

**Affiliations:** 1 Key Laboratory of Experimental Marine Biology, Institute of Oceanology, Chinese Academy of Sciences, Nanhai Road 7, Qingdao 266071, China; lihonglei428@126.com (H.-L.L.); lixmqdio@126.com (X.-M.L.); suiqunyang@163.com (S.-Q.Y.); m8545303@163.com (L.-H.M.); lixin871014@163.com (X.L.); 2 Laboratory of Marine Biology and Biotechnology, Qingdao National Laboratory for Marine Science and Technology, Wenhai Road 1, Qingdao 266237, China; 3 Center for Ocean Mega-Science, Chinese Academy of Sciences, Nanhai Road 7, Qingdao 266071, China

**Keywords:** *Laurencia okamurai*, *Aspergillus terreus*, endophytic fungus, prenylated phenol, benzofuran, antibacterial activity

## Abstract

Three new prenylated phenol derivatives, terreprenphenols A–C (**1**–**3**), along with four known related compounds (**4**–**7**), were isolated from *Aspergillus terreus* EN-539, an endophytic fungus obtained from the marine red alga *Laurencia okamurai*. The structures of these compounds were established by extensive analysis of 1D/2D NMR data, mass spectrometric data, and optical rotation (OR). The corresponding relationship between absolute configuration and optical rotation for known compounds anodendroic acid (**4**) and asperterreusine C (**5**) was ambiguous in literature, and their absolute configurations were therefore discussed and confirmed for the first time by time-dependent density functional (TDDFT) ECD and OR calculations. Compounds **1**–**7** inhibited some common aquatic bacteria with MIC values ranging from 2 to 64 μg/mL.

## 1. Introduction

**Figure 1:**
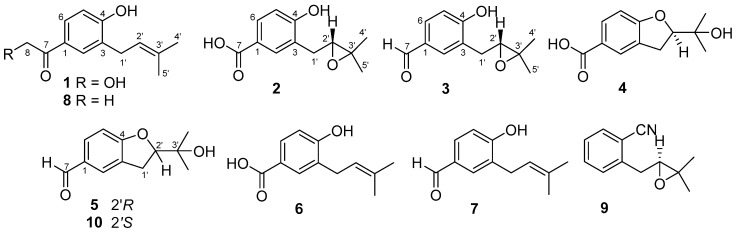
Structures of the isolated compounds **1**–**7** and reference compounds **8**–**10.**

The growing trend in the discovery of new marine natural products from marine microorganisms has continued increasing over the last few years and represented 57% of the total new marine natural products reported in 2017 [1]. However, the rare occurrence of novel compounds and chemical rediscovery have been chronic problems in microbial secondary metabolites research [2,3]. *Aspergillus terreus* is a species complex currently comprised of 14 cryptic species, which is found worldwide in the environment [4]. In our previous research on new bioactive secondary metabolites from marine-derived fungi [5,6,7,8], a fungal strain *Aspergillus terreus* EN-539 was isolated from marine red alga *Laurencia okamurai*, which has been reported as a prolific producer of halogenated organic molecules, such as sesquiterpenes and nonterpenoid C_15_-acetogenins [9]. Chemical investigations were performed on the culture extracts of the marine fungus *A. terreus* EN-539. Two new meroterpenoids, aperterpenes N and O, along with related derivatives have been reported from *A. terreus* EN-539, which was cultured on rice-solid medium [7]. To enhance the chemical diversity of secondary metabolites, the fungal strain *A. terreus* EN-539 was further cultivated on MH2 medium, which resulted in the production of different metabolites compared with that culturing on rice-solid medium, as evidenced by HPLC analysis. As a result, three new prenylated phenol derivatives including terreprenphenol A (**1**), terreprenphenol B (**2**), and terreprenphenol C (**3**), along with four known related compounds (**4**–**7**), were isolated and identified. Moreover, as the corresponding relationship between stereochemistry and optical rotation (OR) for known compounds, anodendroic acid (**4**) and asperterreusine C (**5**), were ambiguous in literature, their absolute configurations were discussed and confirmed on the basis of time-dependent density functional (TDDFT)-ECD and OR calculations. The antimicrobial activities against some common aquatic bacteria, as well as antioxidative activity against DPPH (2,2-diphenyl-1-picrylhydrazyl) radical, were evaluated. This paper describes the isolation, characterization, and bioactivities of compounds **1**–**7** (Figure 1).

## 2. Results and Discussion

### 2.1. Structure Elucidation of the New Compounds

**Figure 2:**
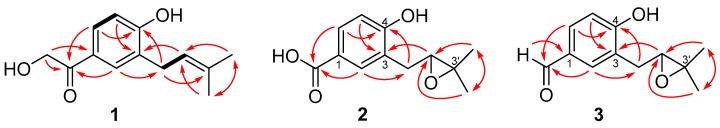
Key ^1^H-^1^H COSY (bold lines) and HMBC (red arrows) correlations of compounds **1**–**3**.

Terreprenphenol A (**1**) was obtained as a colourless solid, and its formula was determined as C_13_H_16_O_3_ on the basis of HRESIMS data (Figure S1), indicating six degrees of unsaturation. The ^1^H and ^13^C NMR spectroscopic data of **1** indicated the presence of a 1,3,4-trisubstituted benzene ring, two methyls, two methylenes (including one oxygenated), one olefinic methine, and two nonprotonated (including one ketone and one olefinic) carbon atoms. Detailed analysis of the ^1^H and ^13^C NMR data (Table 1) revealed that **1** is a prenylated phenol derivative similar to 4-hydroxy-3-(3-methyl-2-butenyl)acetophenone (HMBA, **8**), which is the main secondary metabolite of *Senecio nutans* (Asteraceae), a medicinal plant of northwestern Argentina [10,11]. However, the resonances for one methyl at δ_H_ 2.58 and δ_C_ 26.1 in the ^1^H and ^13^C NMR spectra of HMBA were not present in those of **1**. Instead, signals for an oxygenated methylene were observed at δ_H_ 4.63 (H-8) and δ_C_ 64.3 (C-8) in the ^1^H and ^13^C NMR spectra of **1**. These spectroscopic features suggested that compound **1** was the 8-hydroxylated derivative of HMBA. The COSY and HMBC data (Figure 2) supported the above deduction. The structure of **1** was thus determined and it was named as terreprenphenol A.

Terreprenphenol B (**2**) was obtained as a white, amorphous powder with the molecular formula C_12_H_14_O_4_ as established by HRESIMS data (Figure S7), indicating six degrees of unsaturation. Signals for a 1,3,4-trisubstituted benzene ring were also observed at δ_H_ 6.69 (d, *J* = 8.3 Hz, H-5), 7.70 (d, *J* = 8.3 Hz, H-6), and 7.71 (s, H-2) in the ^1^H NMR spectrum of **2**, indicating that it could be another prenylated phenol derivative. The ^1^H and ^13^C NMR data (Table 1) of **2** were very similar to those of 4-hydroxy-3-prenybenzoic acid (**6**), a known benzoic acid derivative isolated from the dogwood anthracnose fungus *Discula sp.* by Venkatasubbaiah et al. [12]. However, the resonances of two olefinic carbons (δ_C_ 122.8, C-2’; 132.2, C-3’) in the ^13^C NMR spectrum of 4-hydroxy-3-prenybenzoic acid (**6**) were replaced by resonances of two oxygenated carbons (δ_C_ 68.6, C-2’; 76.6, C-3’) in that of **2**. The HRESIMS as well as COSY and HMBC data of **2** suggested that the double bond at C-2’ and C-3’ in **6** was converted into an epoxide ring in **2**.

Compound **2** only possessed one chiral center in epoxidized isopentyl group, similar to that of 2-{[(2*R*)-3,3-dimethyloxiran-2-yl]methyl}benzonitrile (**9**), an intermediate in a cascade approach to cyclic aminonitrones, which has been assigned as 2’*R* configuration on the basis of chemical synthesis and optical rotation [13]. The opposite signs of optical rotations of compound **2** $[{\left[\alpha \right]}_{D}^{25}$ +128.0 (*c* 0.54, CHCl_3_)] and 2-{[(2*R*)-3,3-dimethyloxiran-2-yl]methyl}benzonitrile $[{\left[\alpha \right]}_{D}^{20}$ −65.1 (*c* 1.4, CHCl_3_)] supported the assignement of the absolute configuration of **2** as 2’*S*.

Terreprenphenol C (**3**) was obtained as a colourless solid, and its formula was determined as C_12_H_14_O_3_, with one oxygen atom less than **2**, on the basis of HRESIMS data (Figure S13). Comprehensive analysis of the ^1^H and ^13^C NMR data (Table 1) of **3** showed a structurally close relationship to that of **2**, except that the carbonyl group at C-7 (δ_C_ 173.4) in **2** was replaced by an aldehyde group (δ_C_ 191.9; δ_H_ 10.64, s) in **3**. The planar structure of **3** was further confirmed by the COSY and HMBC data (Figure 2). The absolute configuration of **3** was also determined as 2’*S* on the basis of its optical rotation value of $[{\left[\alpha \right]}_{D}^{25}$ +85.4 (*c* 0.43, CHCl_3_)], compared to that of 2-{[(2*R*)-3,3-dimethyloxiran-2-yl]methyl}benzonitrile (**9**) and **2**. Moreover, it should be mentioned that the benzoate of **3**, methyl 3-((3,3-dimethyloxiran-2-yl)methyl)-4-hydroxybenzoate, was reported as an intermediate in the syntheses of benzofuran derivatives hostmaniene, 5-formyl-2-(isopropyl-1’-ol)benzofuran, and anadenfroic acid, without purification and identification [14].

**Figure 3:**
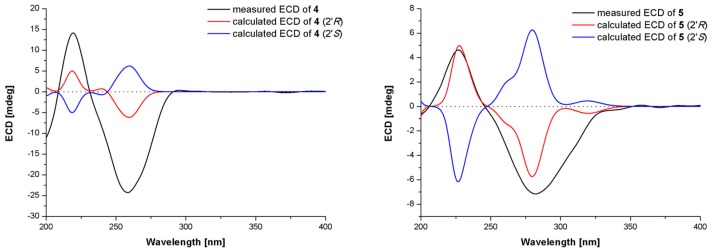
Measured and calculated ECD spectra of compounds **4** and **5** at B3LYP/6-31G level.

Anodendroic acid (**4**) and asperterreusine C (**5**) were isolated and identified by comparing their NMR spectroscopic data (Figures S19–S22) and optical rotations with those reported in the literatures [14,15,16,17]. However, the relationship between absolute configuration and optical rotation for the known compounds anodendroic acid (**4**) and asperterreusine C (**5**) was ambiguous in the literature, and their absolute configurations were therefore discussed and confirmed by TDDFT-ECD and OR calculations. Anodendroic acid (**4**) was first isolated from the higher plant *Anodendron affine* Durce with optical rotation of ${\left[\alpha \right]}_{D}^{26}$ = −19 (*c* 0.7, EtOH) [15], and then its absolute configuration was determined as being *R* by chemical synthesis, with optical rotation of ${\left[\alpha \right]}_{D}^{15}$ = −35.2 (*c* 0.682, EtOH) [18]. Recently, the (+)-*S*-anodendroic acid was isolated and identified from another higher plant *Euodia lepta*, with an optical rotation of ${\left[\alpha \right]}_{D}^{25}$ = +42.0 (*c* 0.15, EtOH) [16]. Quadricinctafuran A, possessing the same planar structure as that of anodendroic acid, was established as having an *R* configuration on the basis of X-ray crystallographic analysis by Prompanya et al. [19]. However, the optical rotation of quadricinctafuran A was measured as ${\left[\alpha \right]}_{D}^{20}$ = +74 (*c* 0.03, MeOH) (*R* configuration with positve OR and *S* configuration with negative OR), inconsistent with the corresponding relationship between sterechemistry and optical rotation (*R* configuration with negative OR, *S* configuration with positive OR) in other literature [14,15,16]. Considering the ambiguity, the absolute configuration of **4** was thus established by the TDDFT-ECD calculation in Gaussian 09 [20]. We obtained the minimum energy conformers by geometry optimization of *S* and *R* isomers of **4**, and then employed the TDDFT method at the B3LYP/6-31G level to obtain a calculated ECD spectra of **4**. The experimental ECD spectrum of **4** exhibited excellent accordance with that calculated for *R* isomer of **4** and was opposite to that calculated for an *S* isomer of **4**, which allowed unambiguous assignment of its absolute configuration (Figure 3). 

Asperterreusine C (**5**) was ever isolated and identified from the marine-derived fungus *Aspergillus terreus* [CFCC 81836] [17] and its absolute configuration was determined as being *R* by comparing the optical rotation ${\left[\alpha \right]}_{D}^{25}$ = −124 (*c* 1.20, MeOH) with that of its enantiomer, (*S*)-5-formyl-2-(isopropyl-1′-ol)-2,3-dihydrobenzofuran (**10**), isolated from the culture broth of *Heterobasidion annosum* $[{\left[\alpha \right]}_{D}^{21}$ = +109.1 (*c* 1.4, CHCl_3_)] [21]. The similar structural characterizations between **4** and **5** encouraged us to confirm the absolute configuration of **5** by using the TDDFT-ECD calculation. The measured ECD spectral behavior (Figure 3) of **5** assigned its absolute configuration as *R* unambiguously. The optical rotations of **4** and **5** were measured as ${\left[\alpha \right]}_{D}^{25}$ = −95.0 (*c* 0.29, MeOH) and ${\left[\alpha \right]}_{D}^{25}$ = −55.0 (*c* 0.51, MeOH), respectively, which satisfied the reported correspondances between sterechemistry and optical rotations (*R* configuration with negative OR and *S* configuration with positive OR) for anodendroic acid [18], (+)-*S*-anodendroic acid [16], asperterreusine C [17], and (*S*)-5-formyl-2-(isopropyl-1′-ol)-2,3-dihydrobenzofuran [21]. This corresponding relationship was further verified by OR calculation at the B3LYP/6-31G(d) level in Gaussian 09 [20]. The OR calculation for compounds **4** and **5** (Table 2) indicated that the *R* configuration was consistent with negative OR and the *S* configuration was featured with the opposite sign, which established the absulute configurations of **4** and **5** by comparing their measured OR values. This matched well with the results of the TDDFT-ECD calculation.

In addition to compounds **1**–**5**, the known prenylated phenol derivatives, 4-hydroxy-3-prenybenzoic acid (**6**) and 4-hydroxy-3-(3-methyl-but-2-enyl)-benzaldehyde (**7**), were isolated and identified from the MH2 culture extract of fungal strain *A. terreus* EN-539. Their structures were established by comparing their NMR data (Figures S23–S26) with those reported in the literature [12,22].

### 2.2. Biological Activities of the Isolated Compounds

Compounds **1**–**7** were assayed for their antioxidative activity against DPPH radical. The result showed that these prenylated phenol and benzofuran derivatives displayed no observed DPPH radical scavenging activity, except that compound **7** exhibited weak scavenging activity with IC_50_ value of 0.9 mM. Butylated hydroxytoluene (BHT) was measured as the positive control for DPPH radical scavenging activity (IC_50_, 72 μM).

Antimicrobial activities of compounds **1**–**7** were also evaluated against human pathogens (*Escherichia coli* and *Staphylococcus aureus*), aquatic bacteria (*Aeromonas hydrophila*, *Edwardsiella tarda*, *Micrococcus luteus*, *Pseudomonas aeruginosa*, *V. harveyi*, *V. parahemolyticus*, *V. vulnificus*), and plant-pathogenic fungi (*Alternaria brassicae*, *Colletotrichum gloeosporioides*, *Fusarium oxysporum*, *Gaeumannomyces graminis*, and *Physalospora piricola*). Compounds **1**, **6**, and **7** showed broad-spectrum inhibitory activity against the pathogenic bacteria in the assay with MIC values ranging from 2 to 64 μg/mL. Compound **1** in particular exhibited potent activity against the aquatic bacteria *A. hydrophila*, *P. aeruginosa*, and *V. harveyi* with MIC values of 9, 9, and 18 μM, respectively. These results (Table 3) indicated that the prenyl group in structures was indispensable for their antibacterial activity (**1**, **6**, **7** vs. **2**–**5**). Compounds **1**–**7** showed no inhibitory activity against the plant-pathogenic fungi in the assay.

## 3. Experimental Section

### 3.1. General Experimental Procedures

Optical rotations were measured on an Optical Activity AA-55 polarimeter (Optical Activity Ltd., Cambridgeshire, UK). UV spectra were measured on a PuXi TU-1810 UV-visible spectrophotometer (Shanghai Lengguang Technology Co. Ltd., Shanghai, China). ECD spectra were acquired on a Chirascan spectropolarimeter (Applied Photophysics Ltd., Leatherhead, UK). The 1D and 2D NMR spectra were obtained at 500 and 125 MHz for ^1^H and ^13^C, respectively, on a Bruker Avance 500 MHz spectrometer (Bruker Biospin Group, Karlsruhe, Germany) with tetramethyl silane (TMS) as an internal standard. Mass spectra were obtained from an API QSTAR Pulsar 1 mass spectrometer (Applied Biosystems, Foster, Waltham, MA, USA). Analytical HPLC analyses were performed using a Dionex HPLC system (Dionex, Sunnyvale, CA, USA) equipped with P680 pump, ASI-100 automated sample injector, and UVD340U multiple wavelength detector controlled by Chromeleon software (version 6.80, Dionex, Sunnyvale, CA, USA). Column chromatography (CC) was performed with silica gel (200–300 mesh, Qingdao Haiyang Chemical Factory, Qingdao, China), Lobar LiChroprep RP-18 (40–60 μm, Merck, Darmstadt, Germany), and Sephadex LH-20 (18–110 μm, Merck). All solvents were distilled prior to use.

### 3.2. Fungal Material

The isolation and identification of the fungal material *A. terreus* EN-539 were the same as those reported in our previous publications [7]. The strain is preserved at the Key Laboratory of Experimental Marine Biology, Institute of Oceanology, Chinese Academy of Sciences (IOCAS).

### 3.3. Fermentation

For chemical investigations, the fresh mycelia of fungal strain *A. terreus* EN-539 was cultured on PDA (Potato Dextrose Agar) medium at 28 ℃ for seven days, and then inoculated into 1 L conical flasks containing 300 mL of MH2 broth medium (sucrose 2%, mannitol 2%, yeast extract 0.3%, peptone 0.5%, K_2_HPO_4_ 0.05%, and MgSO_4_·7H_2_O 0.03% in seawater, which was naturally sourced and filtered from the Huiquan Gulf of the Yellow Sea near the campus of IOCAS, pH 6.5–7.0) for 30 days at room temperature.

### 3.4. Extraction and Isolation

The whole fermented cultures (100 flasks, 30 L) were filtered to separate the broth from the mycelia. The former was extracted four times with ethyl acetate (EtOAc), while the mycelia was extracted four times with a mixture of 80% acetone and 20% H_2_O. The acetone solution was evaporated under reduced pressure to develop an aqueous solution, which was then extracted four times with EtOAc. The two EtOAc solutions were combined and concentrated under reduced pressure to give an extract (48 g), on the basis of TLC and HPLC analysis, which was fractionated by silica gel vacuum liquid chromatography (VLC) using different solvents of increasing polarity from petroleum ether (PE) to MeOH to yield 9 fractions (Frs. 1–9). Fr. 4 (4.5 g), eluted with PE-EtOAc (2:1), was further purified by reversed-phase column chromatography (CC) over Lobar LiChroprep RP-18 with a MeOH-H_2_O gradient (from 10:90 to 100, *v*/*v*) to yield 10 subfractions. Fr 4.2 (MeOH-H_2_O 20:80, 220 mg) was subjected to CC on silica gel (CH_2_Cl_2_-MeOH, from 150:1 to 10:1, *v*/*v*), and then purified by Sephadex LH-20 (MeOH) and preparative TLC (plate: 20 × 20 cm, developing solvents: PE-acetone, 3:1) to obtain compounds **2** (35.6 mg) and **5** (29.1 mg). Fr 4.3 (MeOH-H_2_O 30:70, 330 mg) was fractionated by CC on silica gel eluting with CH_2_Cl_2_-acetone (from 15:1 to 2:1, *v*/*v*) to afford 4 subfractions (Frs. 4.3.1–4.3.4). Fr 4.3.2 was further purified by preparative TLC (plate: 20 × 20 cm, developing solvents: CH_2_Cl_2_-MeOH, 20:1) and CC on Sephadex LH-20 (MeOH) to yield compound **4** (74.0 mg), while Fr 4.3.3 was directly purified by CC on Sephadex LH-20 (MeOH) to yield compound **3** (6.2 mg). Fr. 5 (6.9 g), eluted with PE-EtOAc (1:1), was further purified by reversed-phase CC over Lobar LiChroprep RP-18 with a MeOH-H_2_O gradient (from 10:90 to 100, *v*/*v*) to yield 10 subfractions. Fr 5.5 (MeOH-H_2_O 50:50, 520 mg) was subjected to CC on silica gel (CH_2_Cl_2_-MeOH, from 100:1 to 10:1, *v*/*v*), and then purified by Sephadex LH-20 (MeOH) and preparative TLC (plate: 20 × 20 cm, developing solvents: PE- EtOAc, 1:1) to obtain compound **1** (8.1 mg). Fr 5.5 (MeOH-H_2_O 80:20, 320 mg) was purified by CC on Sephadex LH-20 (MeOH) and then on silica gel (CH_2_Cl_2_-acetone, from 20:1 to 5:1, *v*/*v*) to yield compounds **6** (22.0 mg) and **7** (42.5 mg).

Terreprenphenol A (**1**): Colourless solid; UV (MeOH) *λ*_max_ (log *ε*) 230 (2.32), 297 (2.85) nm; ^1^H and ^13^C NMR data, see Table 1; HRESIMS *m*/*z* 243.0997 [M + Na]^+^ (calcd. for C_13_H_16_NaO_3_, 243.0992).

Terreprenphenol B (**2**): White, amorphous powder; ${\left[\alpha \right]}_{D}^{25}$ +128.0 (*c* 0.54, CHCl_3_); UV (MeOH) *λ*_max_ (log *ε*) 204 (2.57), 250 (2.16) nm; ECD (5.40 mM, MeOH) *λ*_max_ (Δ *ε*) 205 (–1.63), 214 (+1.28), 228 (–0.29), 246 (+0.68) nm; ^1^H and ^13^C NMR data, see Table 1; HRESIMS *m*/*z* 245.0786 [M + Na]^+^ (calcd. for C_12_H_14_NaO_4_, 245.0784).

Terreprenphenol C (**3**): Colourless solid; ${\left[\alpha \right]}_{D}^{25}$ +85.4 (*c* 0.43, CHCl_3_); UV (MeOH) *λ*_max_ (log *ε*) 202 (2.74), 228 (2.56), 287 (2.61) nm; ECD (4.85 mM, MeOH) *λ*_max_ (Δ *ε*) 206 (–1.56), 216 (+0.94), 230 (–0.25), 244 (+0.62) nm;; ^1^H and ^13^C NMR data, see Table 1; HRESIMS *m*/*z* 205.0872 [M – H]^–^ (calcd. for C_12_H_13_O_3_, 205.0870).

### 3.5. Antioxidant and Antimicrobial Assays

Evaluation of compounds **1**–**7** for antioxidative activity against DPPH free radical was carried out using the method described previously [23]. BHT was used as positive control against the DPPH free radical. Antimicrobial activity against human pathogens (*E. coli* EMBLC-1 and *S. aureus* EMBLC-2), aquatic bacteria (*A. hydrophila* QDIO-1, *E. tarda* QDIO-2, *M. luteus* QDIO-3, *P. aeruginosa* QDIO-4, *V. harveyi* QDIO-7, *V. parahemolyticus* QDIO-8, *V. vulnificus* QDIO-10), and plant-pathogenic fungi (*A. brassicae* QDAU-1, *C. gloeosporioides* QDAU-2, *F. oxysporum* QDAU-5, *G. graminis* QDAU-3, and *P. piricolav* QDAU-6), was carried out by the 96-well microtiter plates assay [24]. The pathogens were obtained from the Institute of Oceanology, Chinese Academy of Sciences. Chloramphenicol and amphotericin were used as positive controls for bacteria and fungi, respectively.

### 3.6. Computational Section

Conformational searches were performed via molecular mechanics using the MM+ method in HyperChem software (Version 8.0, Hypercube, Inc., Gainesville, FL, USA), and the geometries were further optimized at the B3LYP/6-31G(d) level via Gaussian 09 software (Version D.01; Gaussian, Inc.:Wallingford, CT, USA) [20] to give the energy-minimized conformers. Then, the optimized conformers were subjected to the calculations of ECD and OR by using TDDFT at B3LYP/6-31G level. Solvent effects of the MeOH solution were evaluated at the same DFT level using the SCRF/PCM method.

## 4. Conclusions

In summary, chemical investigations were performed on the marine fungus *A. terreus* EN-539. Two new meroterpenoids, aperterpenes N and O, along with related derivatives have been reported from *A. terreus* EN-539, which was cultured on rice-solid medium [7]. The coculture of *A. terreus* EN-539 and the symbiotic fungus *Paecilomyces lilacinus* EN-531 induced the production of a new terrein derivative, namely asperterrein and a known dihydroterrein, which were not detected in the axenic cultures of both strains [25]. To enhance the chemical diversity of secondary metabolites, *A. terreus* EN-539 was further cultivated on MH2 medium, which resulted in the production of three new prenylated phenol derivatives including terreprenphenol A (**1**), terreprenphenol B (**2**), and terreprenphenol C (**3**), along with four known related compounds (**4**–**7**). The absolute configurations of benzofuran derivatives (**4** and **5**) were discussed and confirmed on the basis of TDDFT-ECD and OR calculations. Compounds **1**, **6**, and **7** showed broad-spectrum inhibitory activity against the pathogenic bacteria in the assay with MIC values ranging from 2 to 64 μg/mL, which might be used as potential molecules in the development of drug leads, or modified to find more active derivatives for the treatment of microbial infection in aquaculture.

## Figures and Tables

**Table 1:** ^1^H (500 MHz) and ^13^C (125 MHz) NMR data of compounds **1**–**3**.

No.	1 *^a^*		2 *^b^*		3 *^a^*	
	δ_C_	δ_H_ (*J* in Hz)	δ_C_	δ_H_ (*J* in Hz)	δ_C_	δ_H_ (*J* in Hz)
1	128.1, C		129.1, C		129.8, C	
2	128.9, CH	7.59, s	130.6, CH	7.71, s	133.2, CH	8.50, s
3	125.8, C		118.2, C		121.8, C	
4	160.8, C		154.5, C		159.2, C	
5	115.0, CH	6.77, d (9.1)	115.1, CH	6.69, d (8.3)	117.9, CH	7.72, d (8.4)
6	127.7, CH	7.58, d (9.1)	128.0, CH	7.70, d (8.3)	129.8, CH	8.46, d (8.4)
7	196.4, C		173.4, C		191.9, CH	10.64, s
8	64.3, CH_2_	4.63, s				
1’	28.1, CH_2_		30.3, CH_2_	2.75, dd (7.4, 16.6) 3.03, dd (5.3, 16.6)	31.4, CH_2_	3.54, dd (7.1, 16.8) 3.85, dd (4.9, 16.8)
2’	122.7, CH	3.22, d (7.3)	68.6, CH	3.77, dd (5.3, 7.4)	68.0, CH	4.54, dd (4.9, 7.1)
3’	131.3, C	5.29, t (7.3)	76.6, C		79.4, C	
4’	17.6, CH_3_	1.67, s	19.2, CH_3_	1.26, s	22.0, CH_3_	2.07, s
5’	25.5, CH_3_	1.70, s	24.1, CH_3_	1.33, s	26.2, CH_3_	2.13, s

*^a^* Measured in DMSO-*d*_6_. *^b^* Measured in CD_3_OD.

**Table 2:** The calculated and measured optical rotations (ORs) of compounds **4** and **5** ^a^.

Compounds	4		5	
	2’*R*	2’*S*	2’*R*	2’*S*
calculated OR	−60.9	+61.0	−91.3	+95.5
measured OR	−95.0		−55.0	

*^a^* The ORs were calculated and measured at 589.3 nm in MeOH.

**Table 3:** The antimicrobial activities of compounds **1**–**7** (MIC, μg/mL) ^a^.

Compounds	*Ah*	*Et*	*Ec*	*Ml*	*Pa*	*Sa*	*Vh*	*Vp*	*Vv*
1	2	32	32	16	2	8	4	8	32
2	64	n.a.	32	32	64	n.a.	n.a.	n.a.	n.a.
3	n.a.	n.a.	64	32	n.a.	n.a.	n.a.	n.a.	n.a.
4	n.a.	32	n.a.	n.a.	n.a.	64	n.a.	n.a.	n.a.
5	n.a.	64	n.a.	32	n.a.	32	n.a.	n.a.	n.a.
6	8	16	16	n.a.	n.a.	64	32	8	n.a.
7	4	16	32	8	16	16	8	8	64
Chloramphenicol	1	0.5	2	2	1	2	2	4	4

^a^*Ah*: *Aeromonas hydrophila*. *Et*: *Edwardsiella tarda*. *Ec*: *Escherichia coli*. *Ml*: *Micrococcus luteus*. *Pa*: *Pseudomonas aeruginosa*. *Sa*: *Staphylococcus aureus*. *Vh*: *Vibrio harveyi*. *Vp*: *V. parahemolyticus*. *Vv*: *Vibrio vulnificus*. n.a.: No activity, MIC > 64 μg/mL.

## Supplementary Materials

The following are available online at https://www.mdpi.com/1660-3397/17/11/605/s1: The HRESIMS, 1D and 2D NMR spectra of compounds **1**–**3**, as well as the ^1^H and ^13^C NMR spectra of compounds **4**–**7**.
