# Supplementary material for: Prenylated Phenol and Benzofuran Derivatives from Aspergillus terreus EN-539, an Endophytic Fungus Derived from Marine Red Alga Laurencia okamurai
Source: Mar Drugs. 2019 Oct 24;17(11):605. doi: 10.3390/md17110605 (PMC6891482; doi:10.3390/md17110605)
Supplement: Supplementary file 1 [file marinedrugs-17-00605-s001.pdf]

Supplementary Material

Prenylated Phenol and Benzofuran Derivatives from *Aspergillus terreus* EN-539, an Endophytic Fungus Derived from Marine Red Alga *Laurencia okamurai*

Hong-Lei Li [1,2,3], Xiao-Ming Li [1,2,3], Sui-Qun Yang [1,2,3], Ling-Hong Meng [1,2,3], Xin Li [1,2,3] and Bin-Gui Wang [1,2,3*]

[1] Key Laboratory of Experimental Marine Biology, Institute of Oceanology, Chinese Academy of Sciences, Nanhai Road 7, Qingdao 266071, China; E-Mails: lihonglei428@126.com (H.-L.L.); lixmqdio@126.com (X.-M.L.); suiqunyang@163.com (S.-Q.Y.); m8545303@163.com (L.-H.M.); lixin871014@163.com (X.L.)

[2] Laboratory of Marine Biology and Biotechnology, Qingdao National Laboratory for Marine Science and Technology, Wenhai Road 1, Qingdao 266237, China

[3] Center for Ocean Mega-Science, Chinese Academy of Sciences, Nanhai Road 7, Qingdao 266071, China

***** Correspondence: wangbg@ms.qdio.ac.cn (B.-G.W.); Tel: +86-532-8289-8553 (B.-G.W.)

Content

Fig. S1 HRESIMS spectrum of compound **1**.
Fig. S2 ^{1}H NMR (500 MHz, DMSO-d_6) of compound **1**.
Fig. S3 ^{13}C NMR and DEPT (125 MHz, DMSO-d_6) of compound **1**.
Fig. S4 COSY spectrum of compound **1**.
Fig. S5 HSQC spectrum of compound **1**.
Fig. S6 HMBC spectrum of compound **1**.
Fig. S7 HRESIMS spectrum of compound **2**.
Fig. S8 ^{1}H NMR (500 MHz, CD_3OD) of compound **2**.
Fig. S9 ^{13}C NMR and DEPT (125 MHz, CD_3OD) of compound **2**.
Fig. S10 COSY spectrum of compound **2**.
Fig. S11 HSQC spectrum of compound **2**.
Fig. S12 HMBC spectrum of compound **2**.
Fig. S13 HRESIMS spectrum of compound **3**.
Fig. S14 ^{1}H NMR (500 MHz, DMSO-d_6) of compound **3**.
Fig. S15 ^{13}C NMR and DEPT (125 MHz, DMSO-d_6) of compound **3**.
Fig. S16 COSY spectrum of compound **3**.
Fig. S17 HSQC spectrum of compound **3**.
Fig. S18 HMBC spectrum of compound **3**.
Fig. S19 ^{1}H NMR (500 MHz, DMSO-d_6) of compound **4**.
Fig. S20 ^{13}C NMR and DEPT (125 MHz, DMSO-d_6) of compound **4**.
Fig. S21 ^{1}H NMR (500 MHz, DMSO-d_6) of compound **5**.
Fig. S22 ^{13}C NMR and DEPT (125 MHz, DMSO-d_6) of compound **5**.
Fig. S23 ^{1}H NMR (500 MHz, DMSO-d_6) of compound **6**.
Fig. S24 ^{13}C NMR and DEPT (125 MHz, DMSO-d_6) of compound **6**.
Fig. S25 ^{1}H NMR (500 MHz, DMSO-d_6) of compound **7**.
Fig. S26 ^{13}C NMR and DEPT (125 MHz, DMSO-d_6) of compound **7**.
Fig. S27 DFT optimized conformers and populations of compound **4** (*S*) above 2% population.
Fig. S28 DFT optimized conformers and populations of compound **4** (*R*) above 2% population.
Fig. S29 DFT optimized conformers and populations of compound **5** (*S*)above 2% population.
Fig. S30 DFT optimized conformers and populations of compound **5** (*R*) above 2% population.

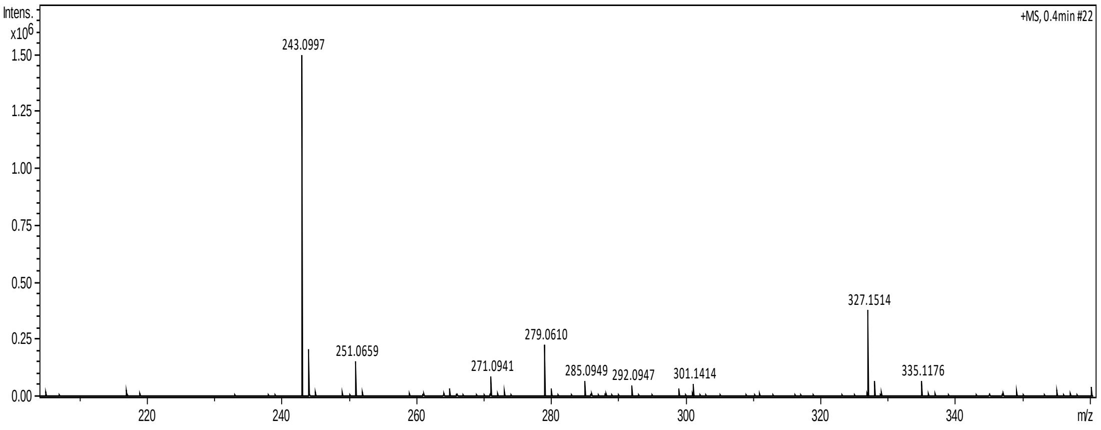

Fig. S1 HRESIMS spectrum of compound **1**.

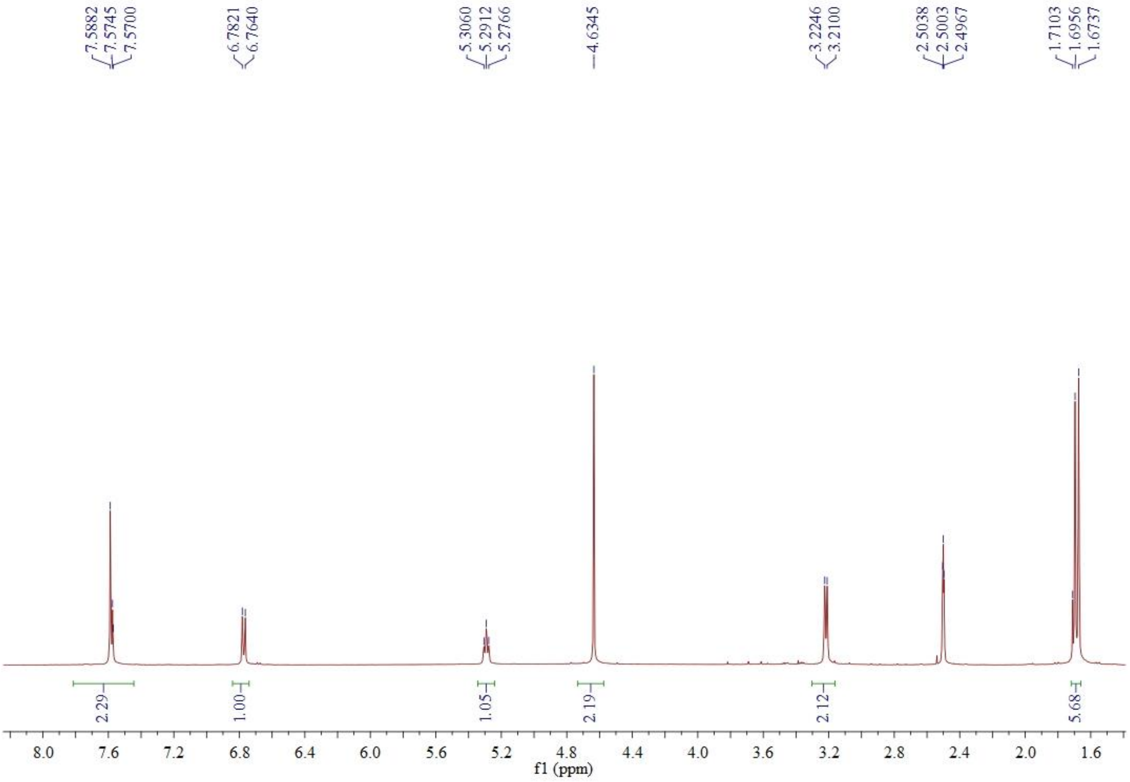

Fig. S2 ^{1}H NMR (500 MHz, DMSO-d_6) of compound **1**.

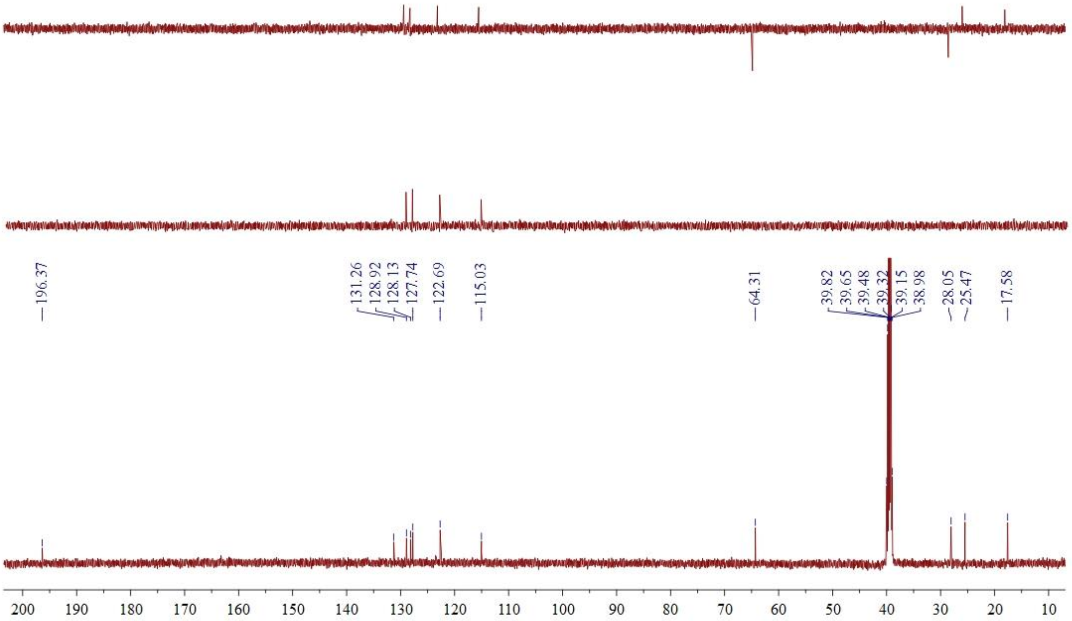

Fig. S3 ^{13}C NMR and DEPT (125 MHz, DMSO-d_6) of compound **1**.

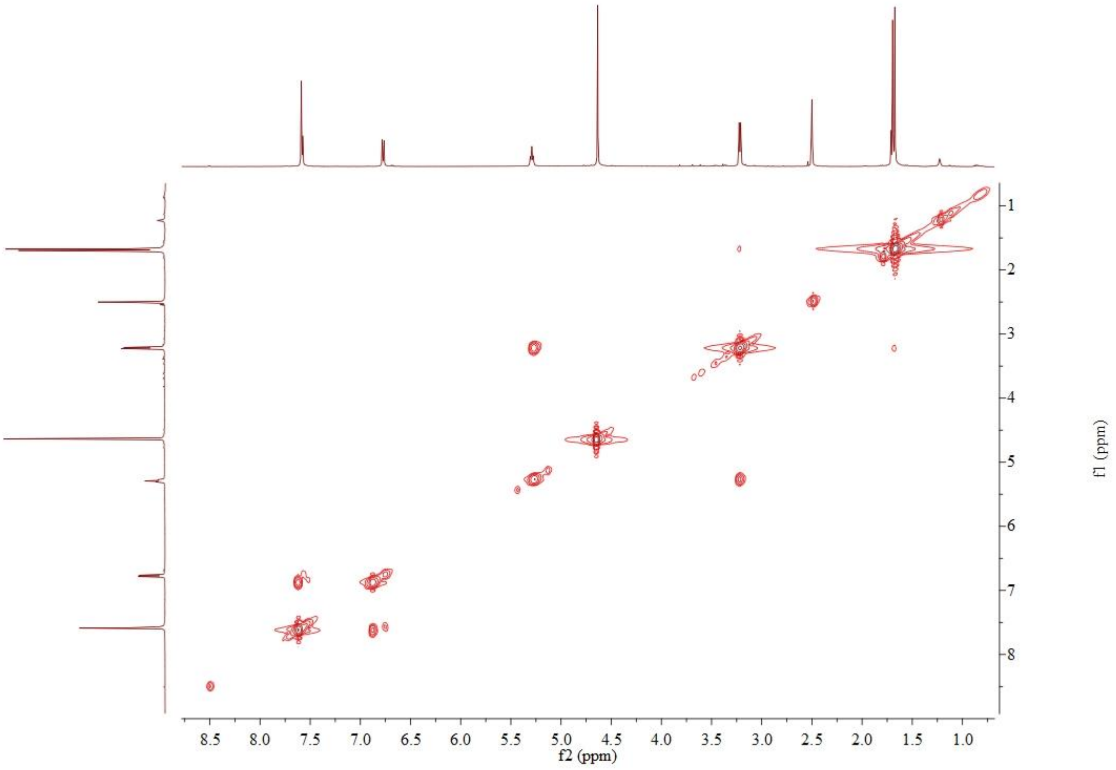

Fig. S4 COSY spectrum of compound **1**.

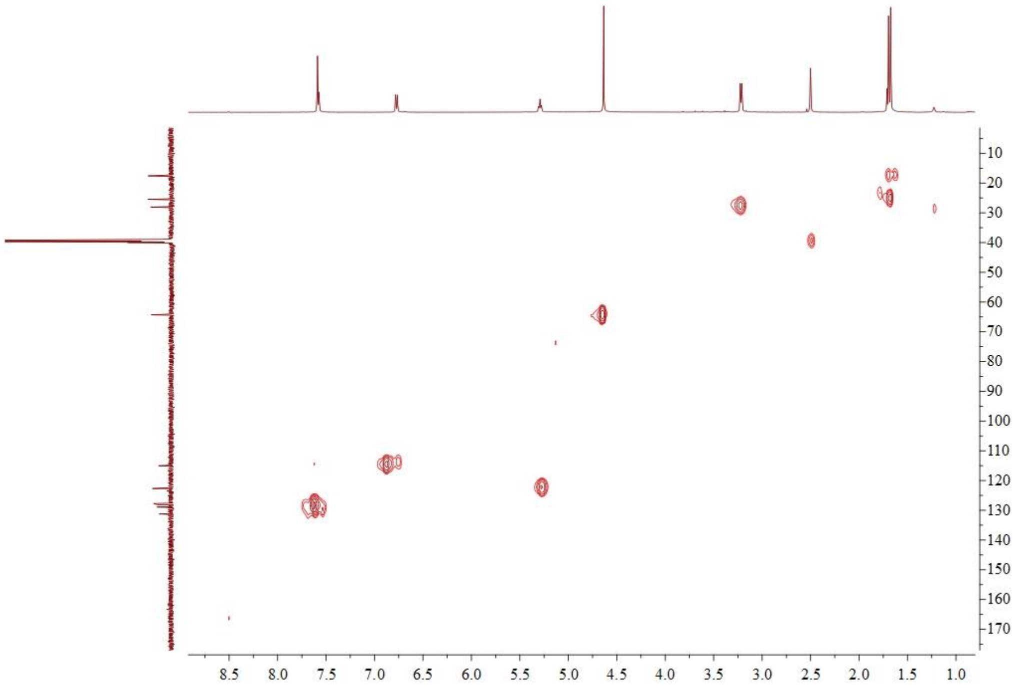

Fig. S5 HSQC spectrum of compound **1**.

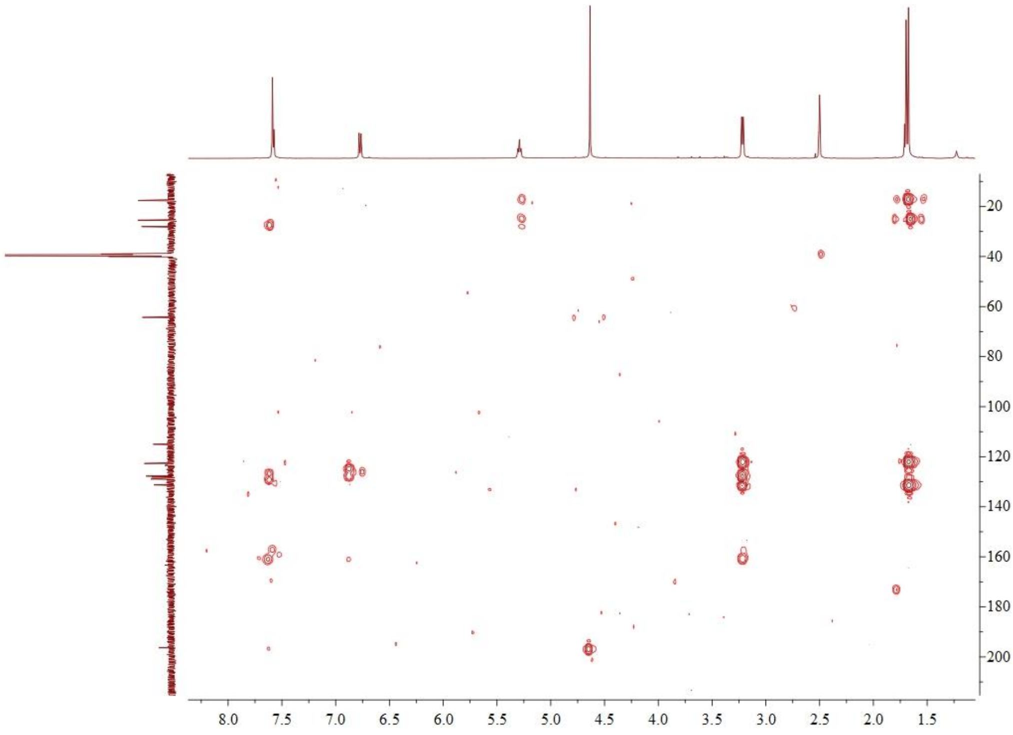

Fig. S6 HMBC spectrum of compound **1**.

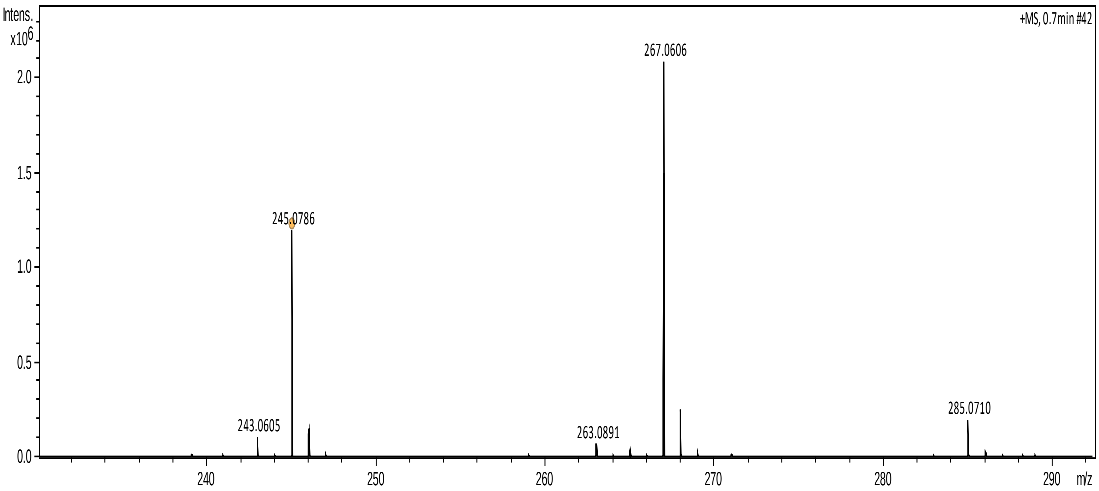

Fig. S7 HRESIMS spectrum of compound **2**.

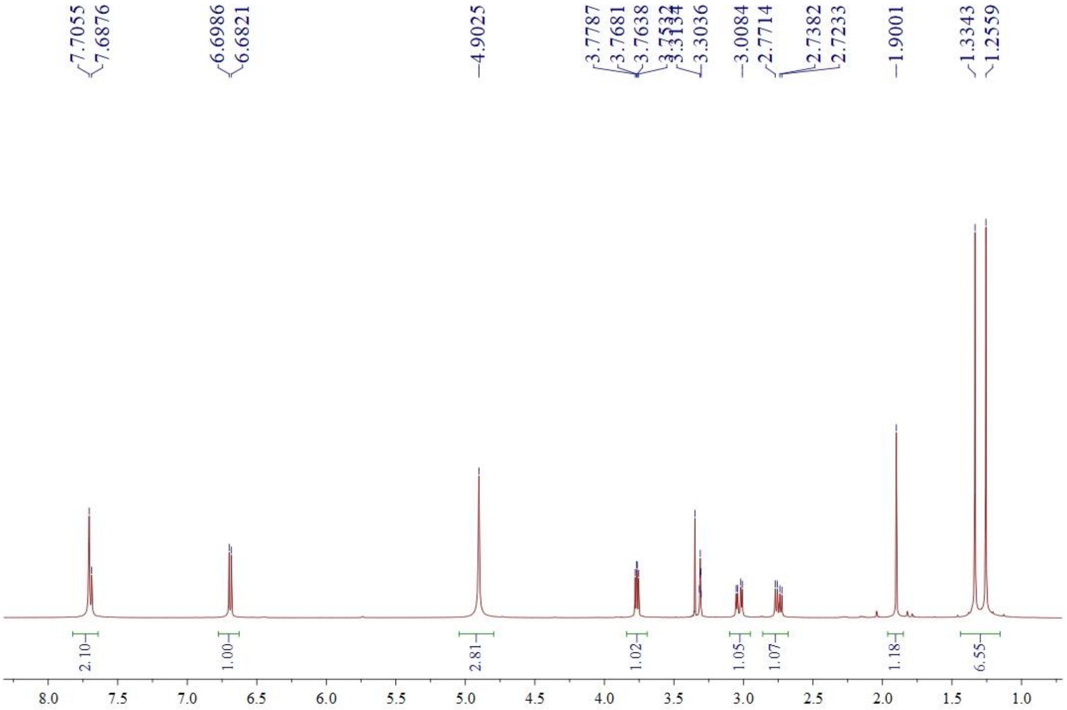

Fig. S8 ^{1}H NMR (500 MHz, CD_3OD) of compound **2**.

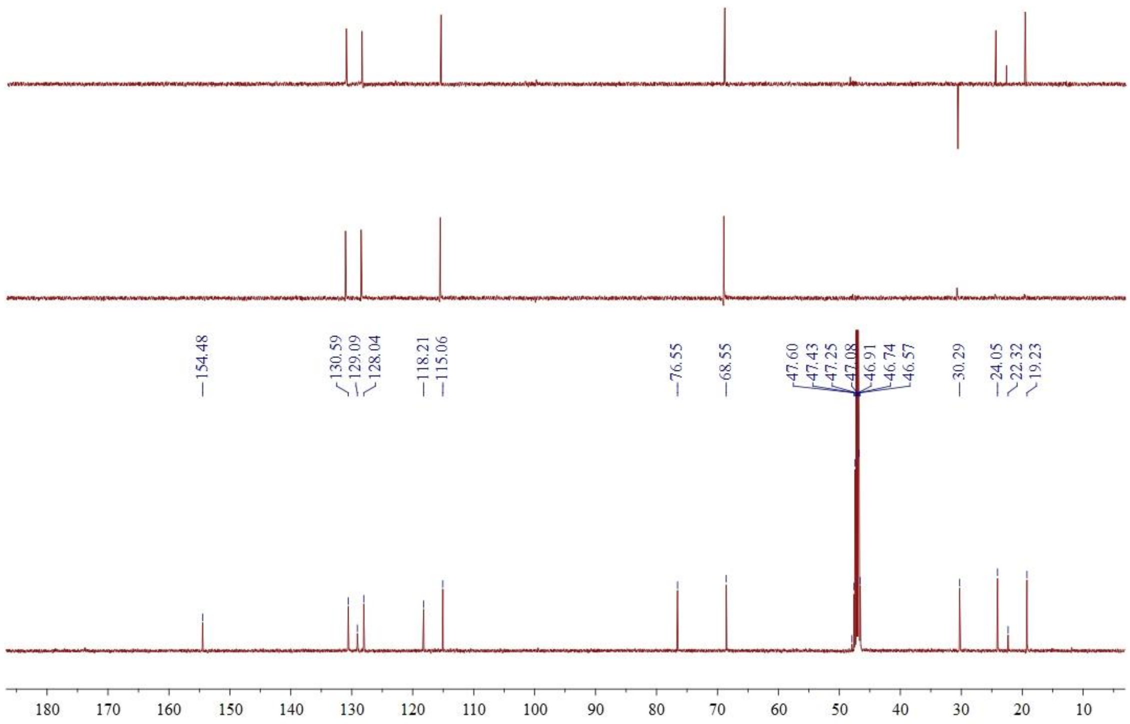

Fig. S9 ^{13}C NMR and DEPT (125 MHz, CD_3OD) of compound **2**.

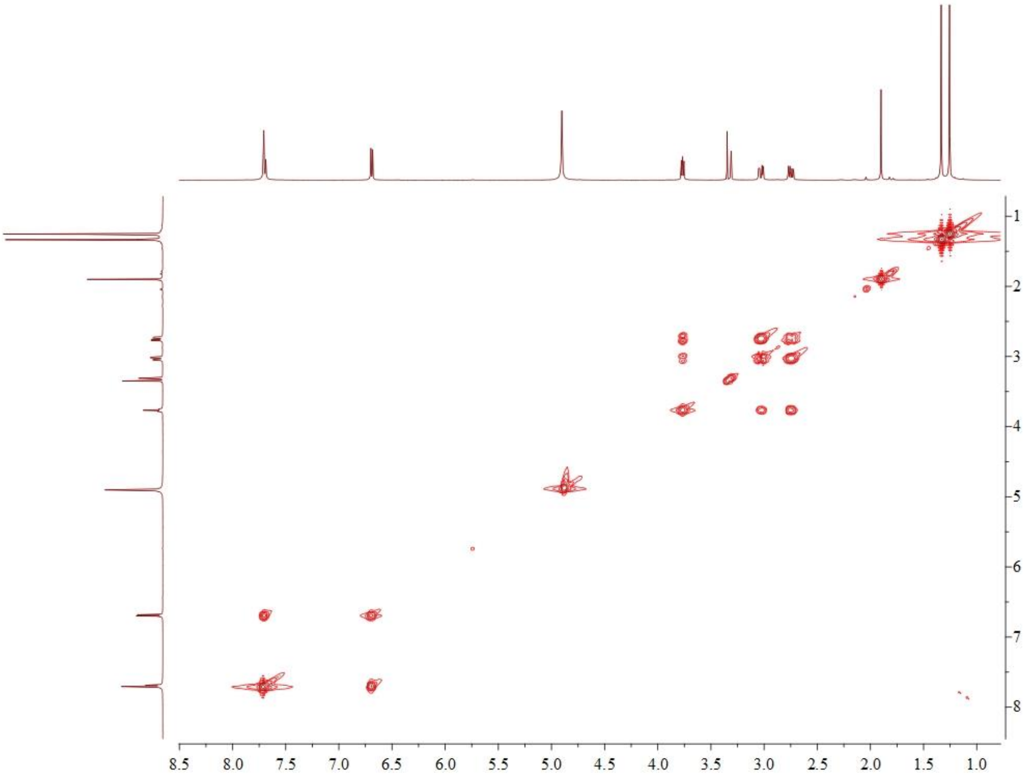

Fig. S10 COSY spectrum of compound **2**.

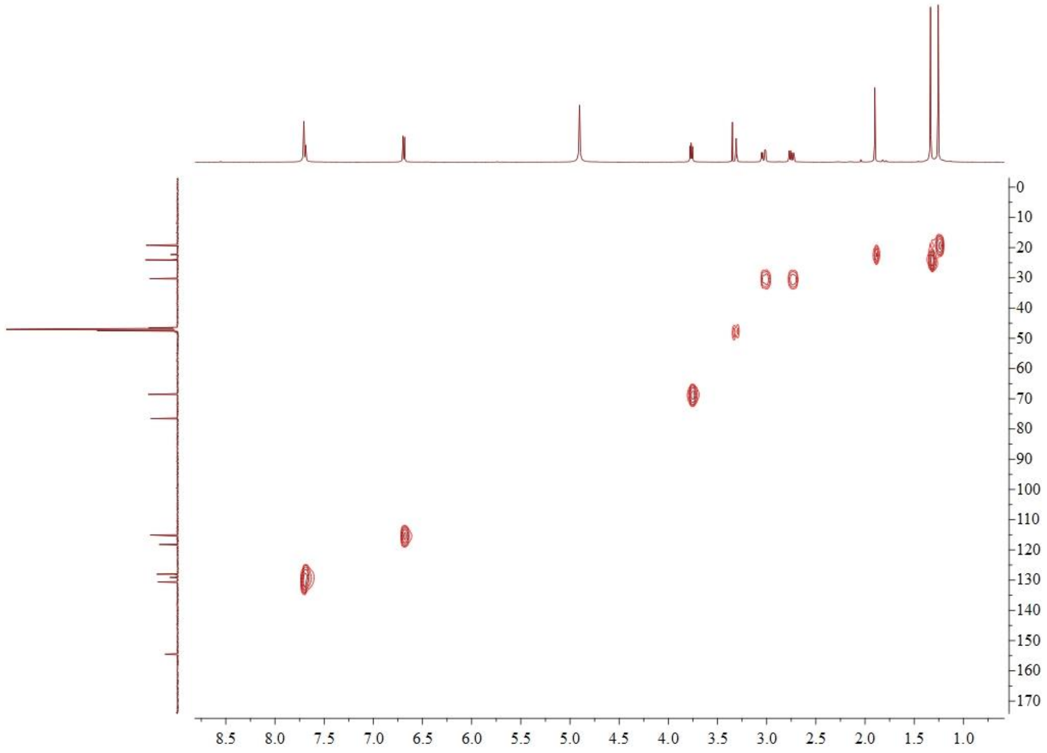

Fig. S11 HSQC spectrum of compound **2**.

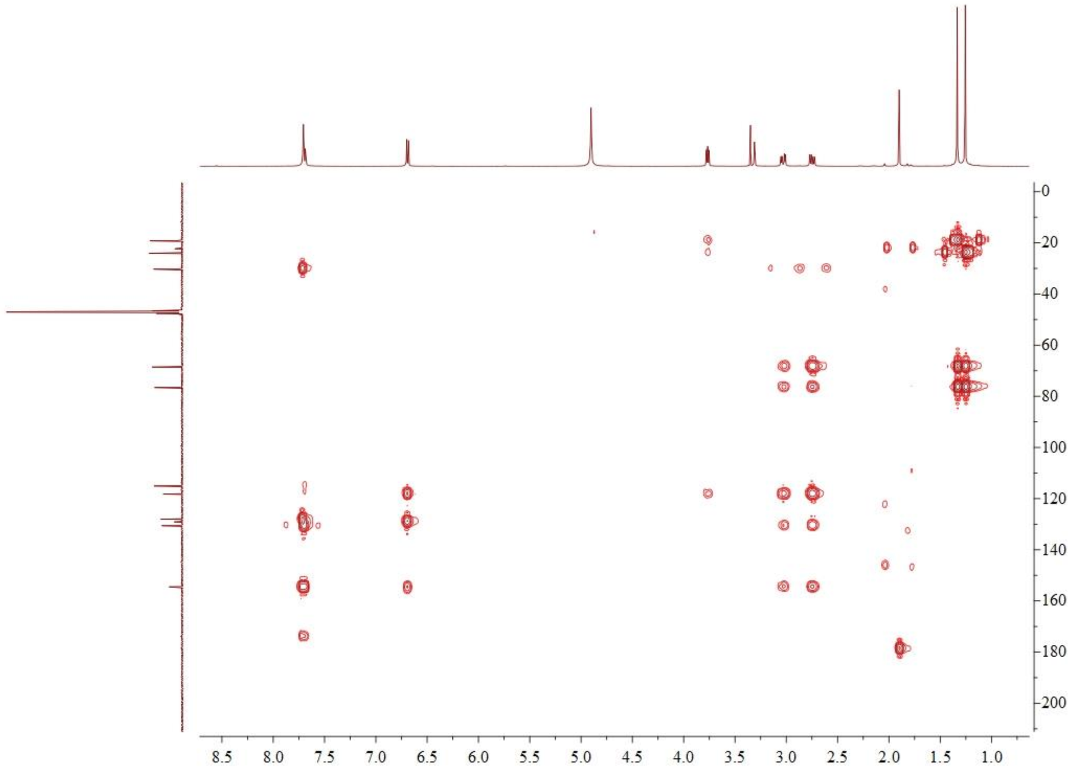

Fig. S12 HMBC spectrum of compound **2**.

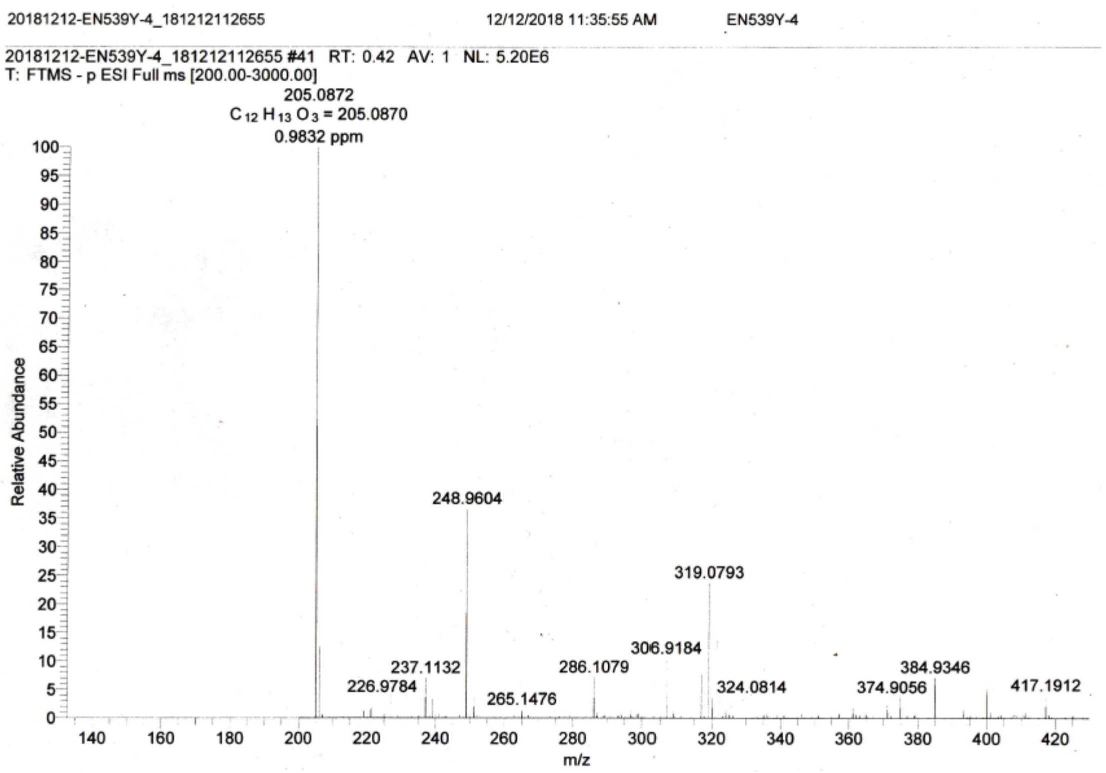

Fig. S13 HRESIMS spectrum of compound **3**.

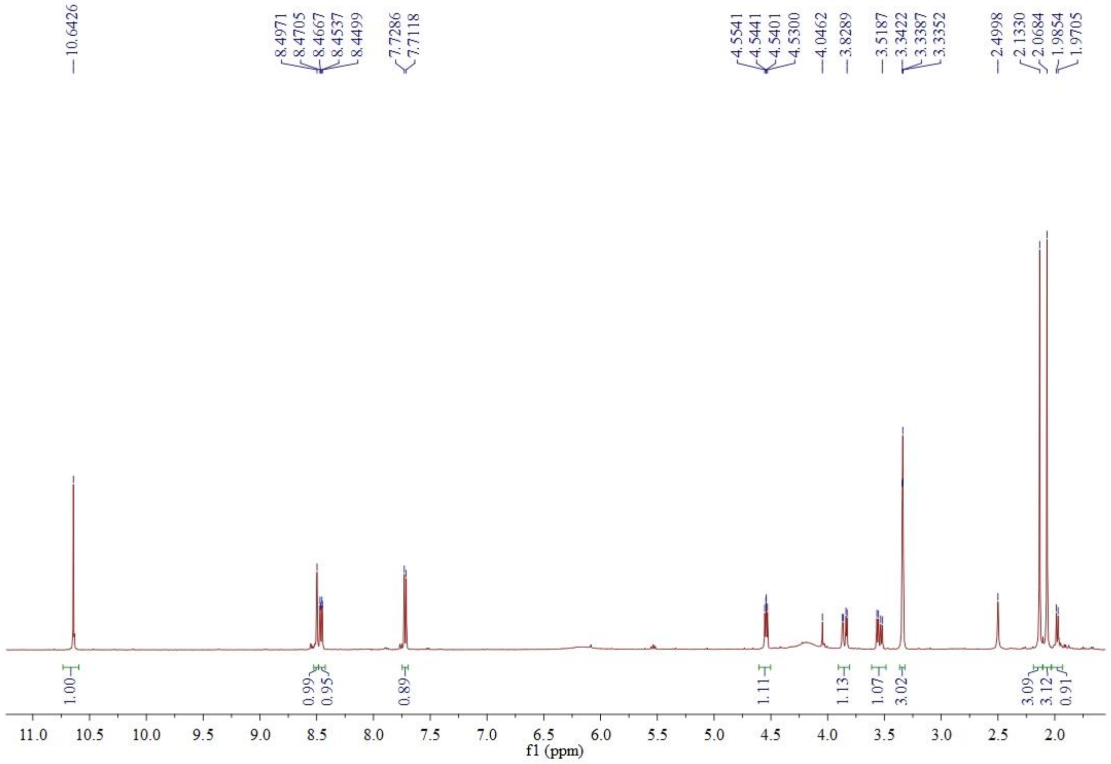

Fig. S14 ^{1}H NMR (500 MHz, DMSO-d_6) of compound **3**.

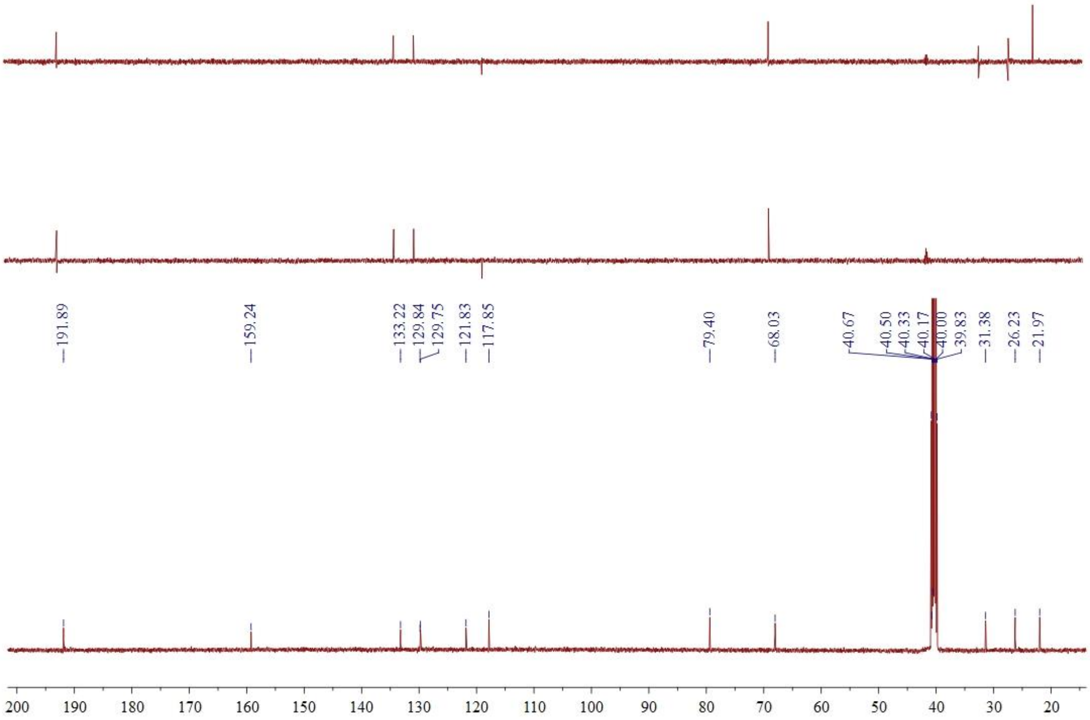

Fig. S15 ^{13}C NMR and DEPT (125 MHz, DMSO-d_6) of compound **3**.

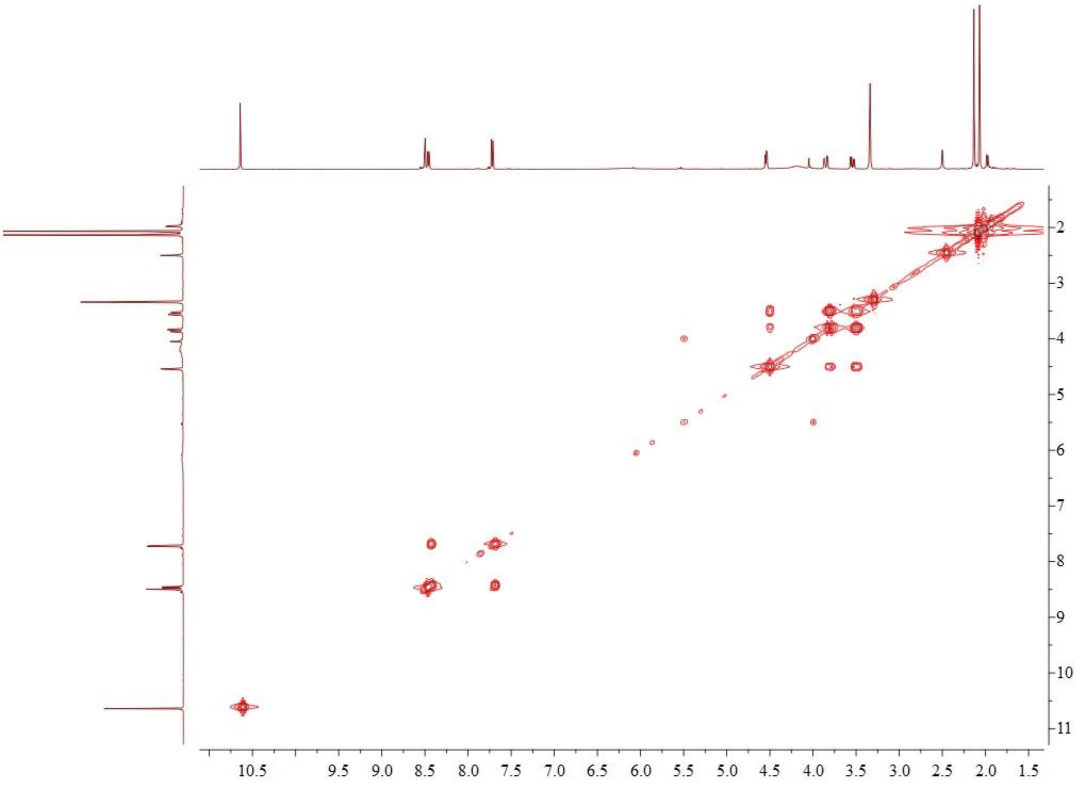

Fig. S16 COSY spectrum of compound **3**.

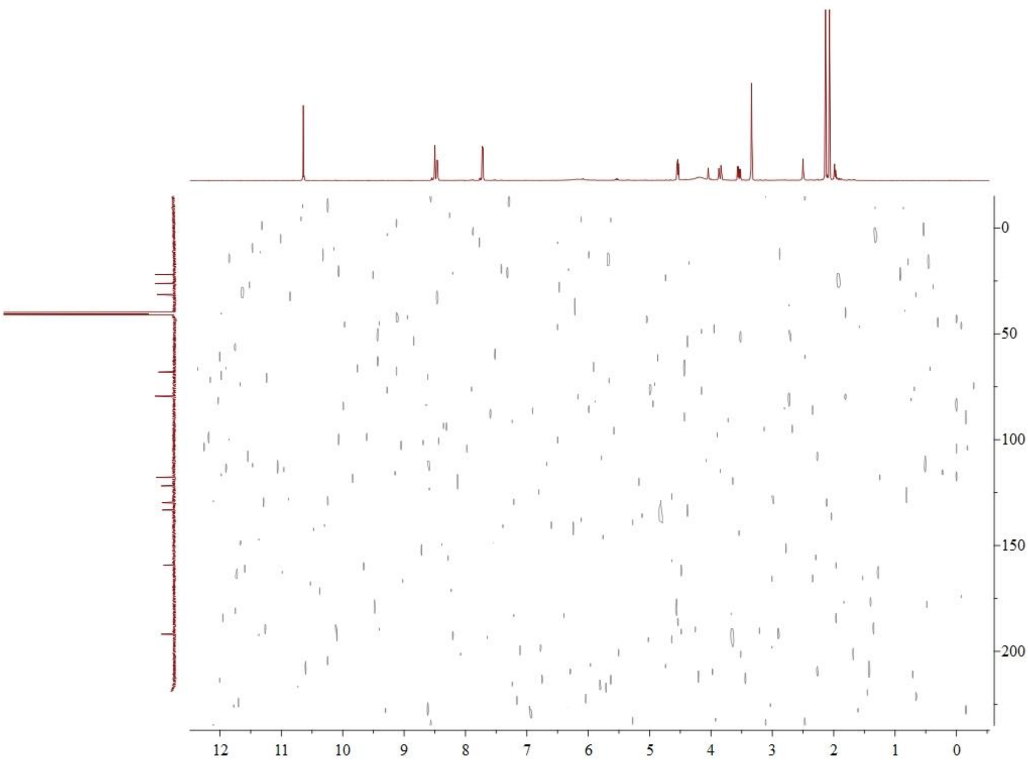

Fig. S17 HSQC spectrum of compound **3**.

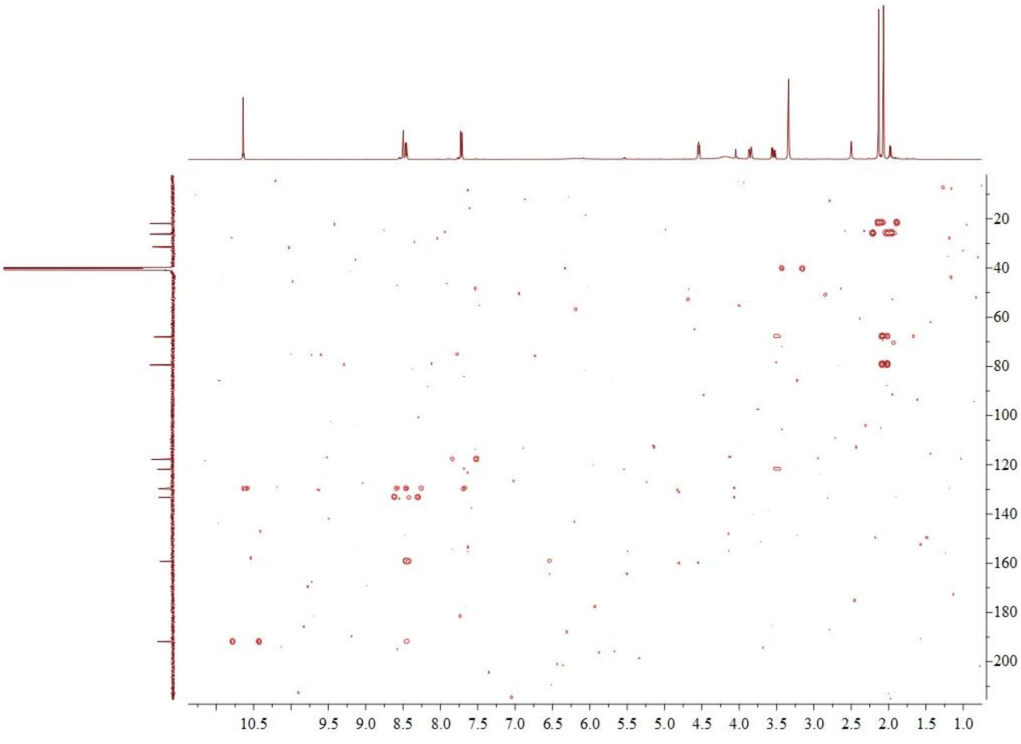

Fig. S18 HMBC spectrum of compound **3**.

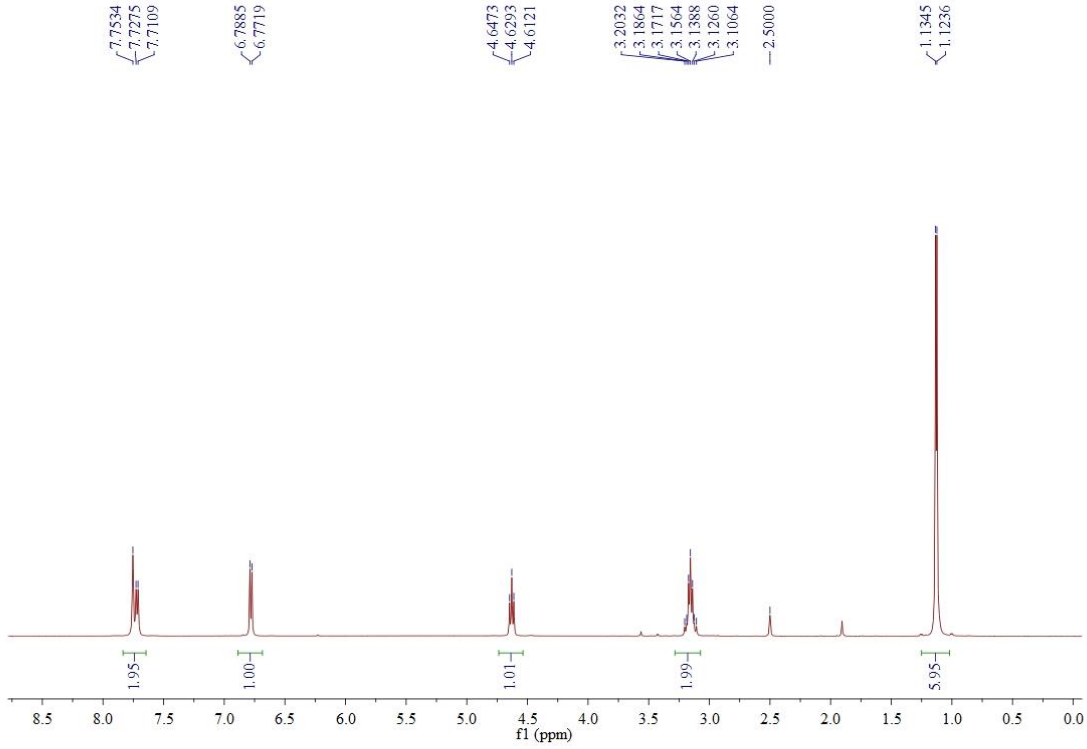

Fig. S19 ^{1}H NMR (500 MHz, DMSO-d_6) of compound **4**.

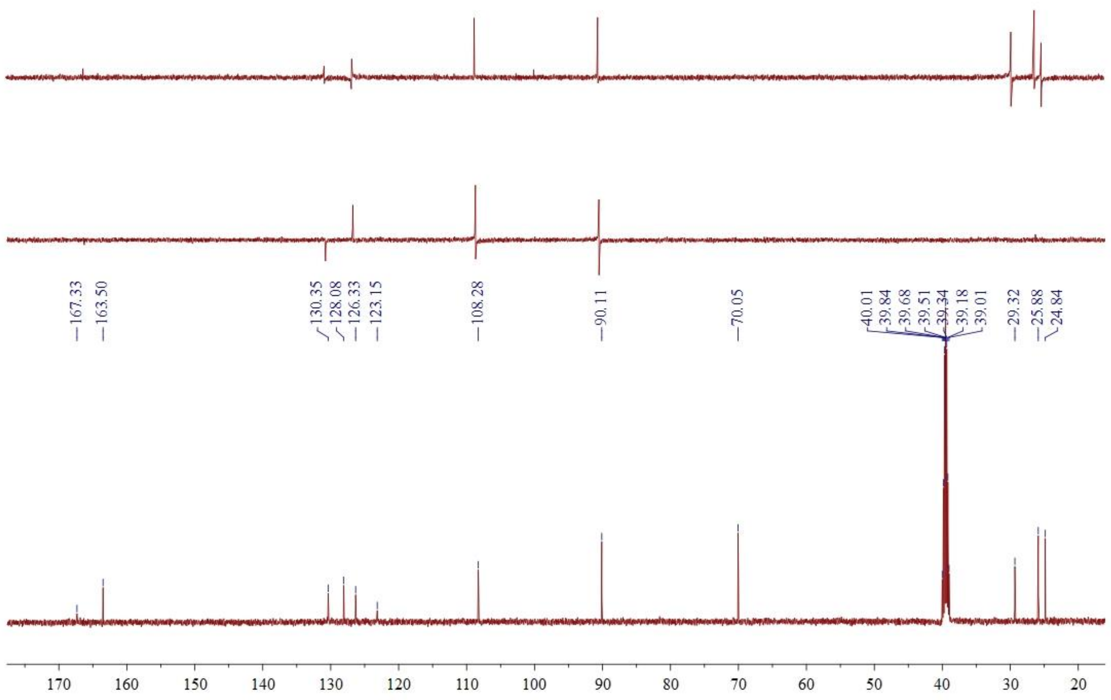

Fig. S20 ^{13}C NMR and DEPT (125 MHz, DMSO-d_6) of compound **4**

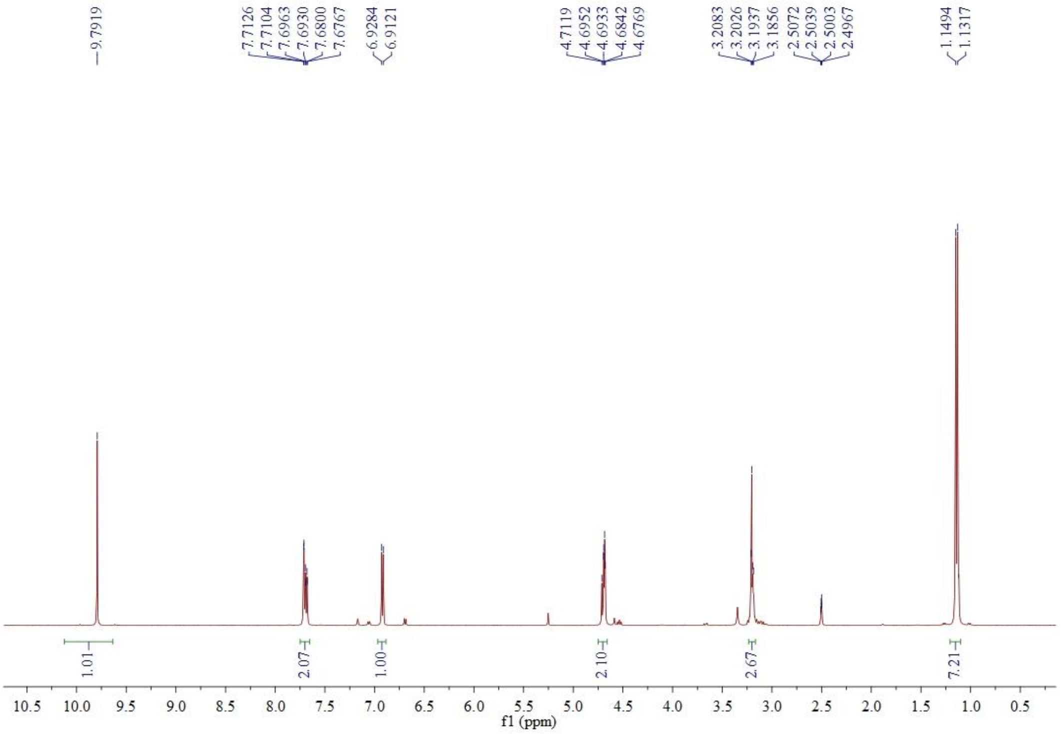

Fig. S21 ^{1}H NMR (500 MHz, DMSO-d_6) of compound **5**.

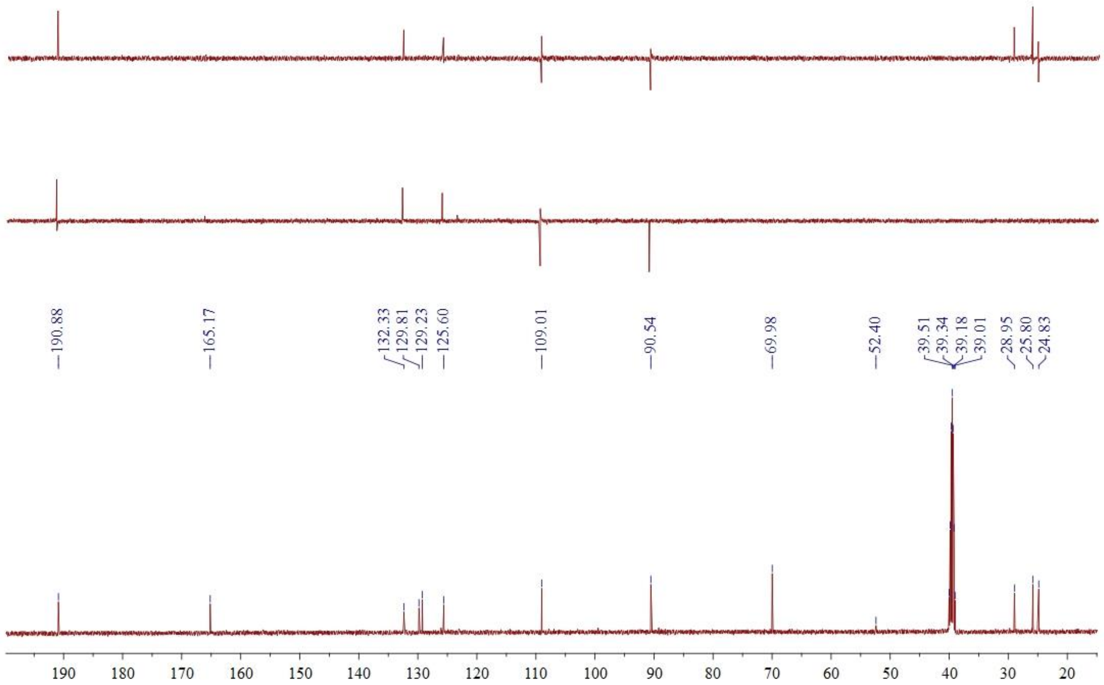

Fig. S22 ^{13}C NMR and DEPT (125 MHz, DMSO-d_6) of compound **5**.

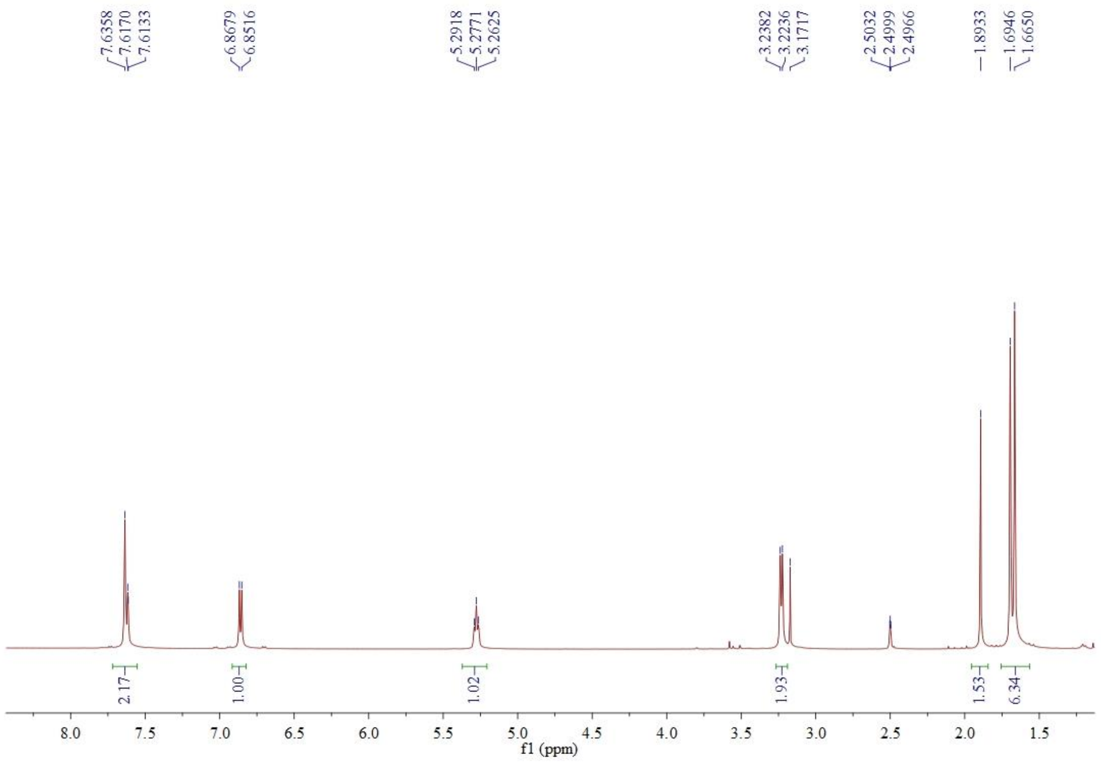

Fig. S23 ^{1}H NMR (500 MHz, DMSO-d_6) of compound **6**.

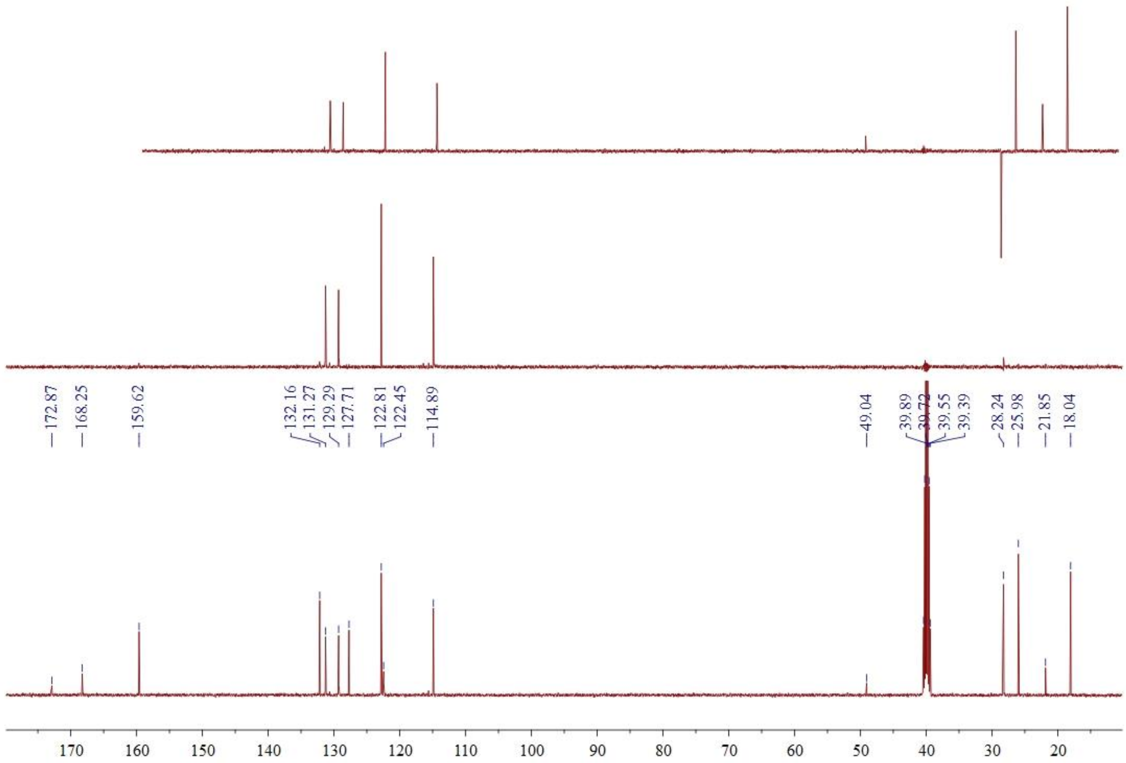

Fig. S24 ^{13}C NMR and DEPT (125 MHz, DMSO-d_6) of compound **6**.

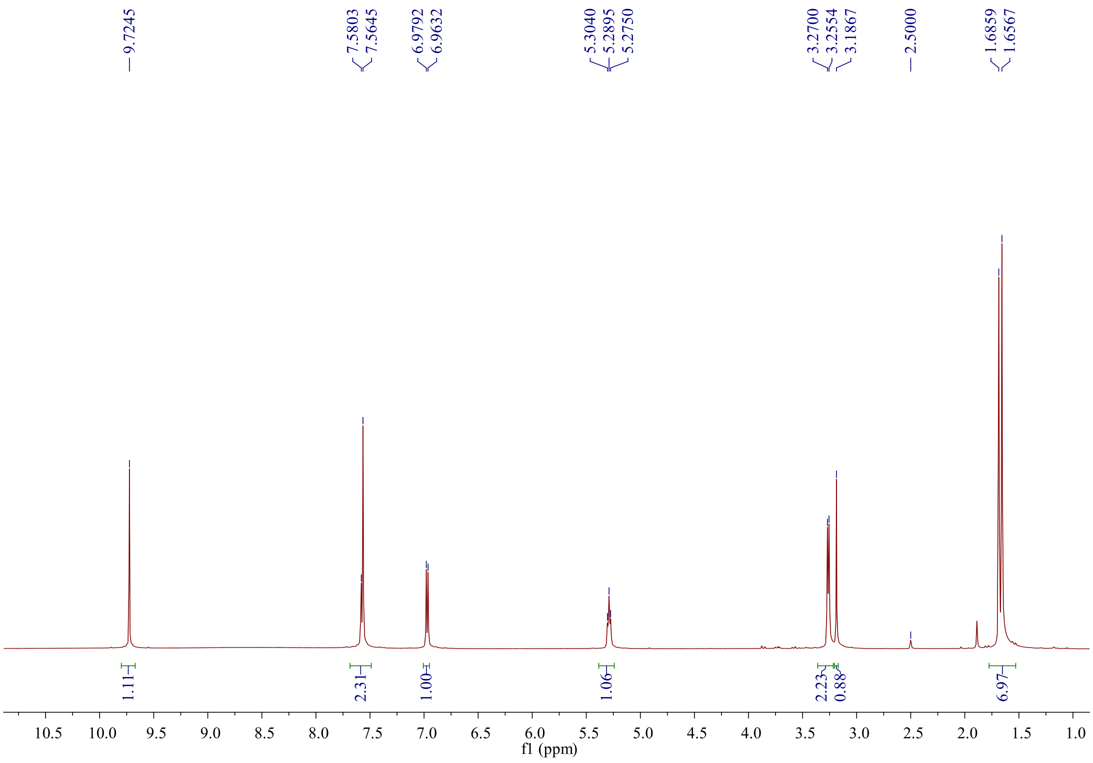

Fig. S25 ^{1}H NMR (500 MHz, DMSO-d_6) of compound **7**.

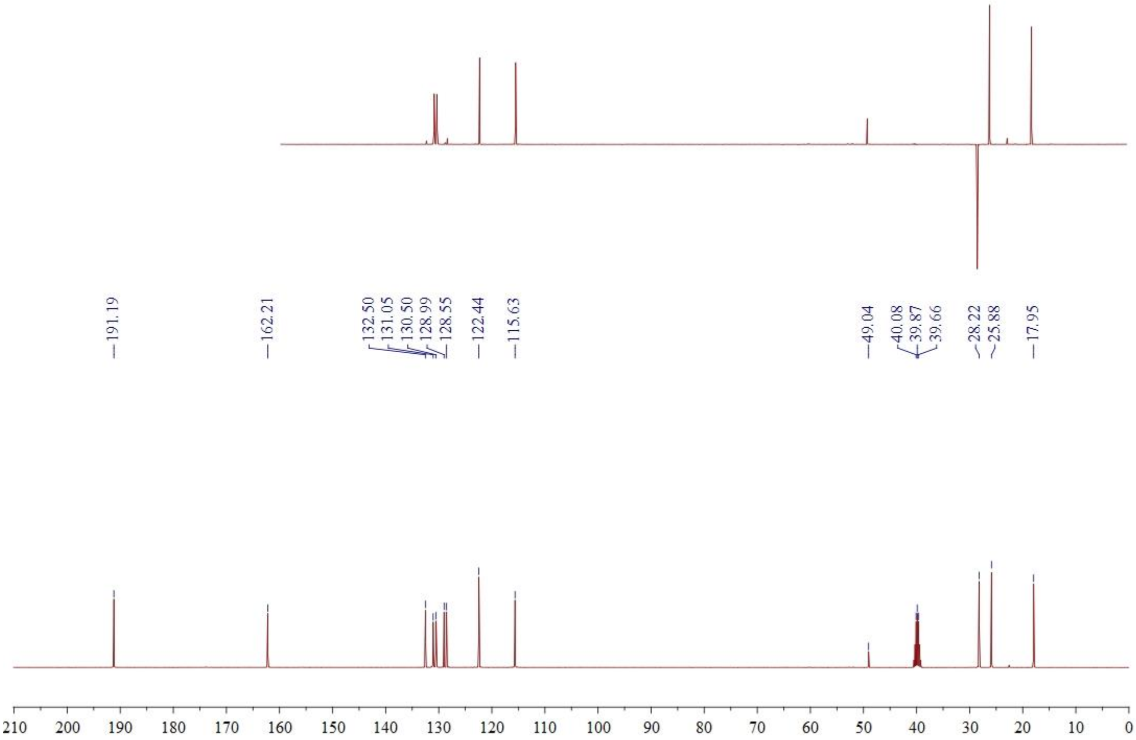

Fig. S26 ^{13}C NMR and DEPT (125 MHz, DMSO-d_6) of compound **7**.

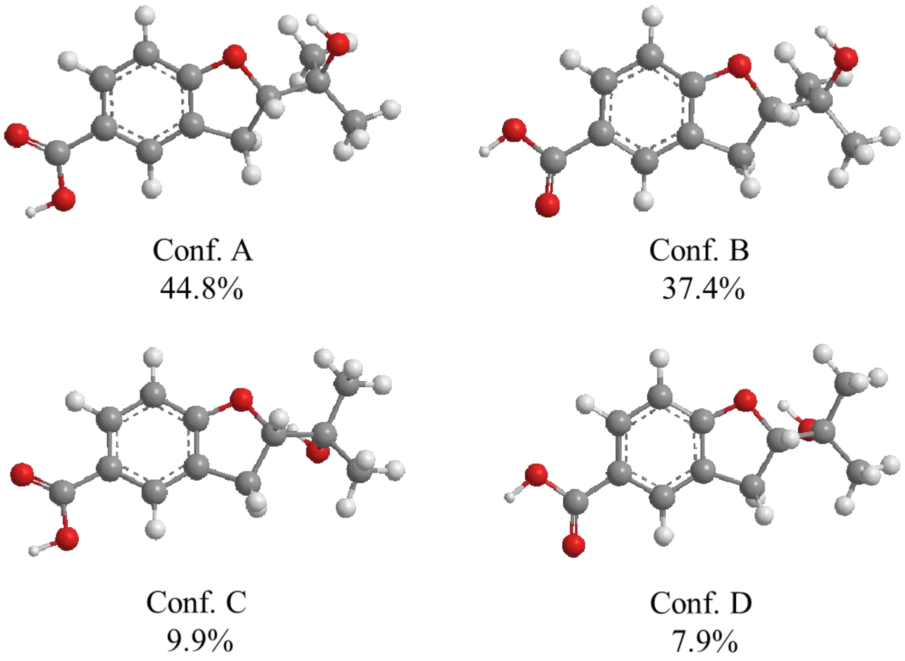

Fig. S27 DFT optimized conformers and populations of compound **4** (*S*) above 2% population.

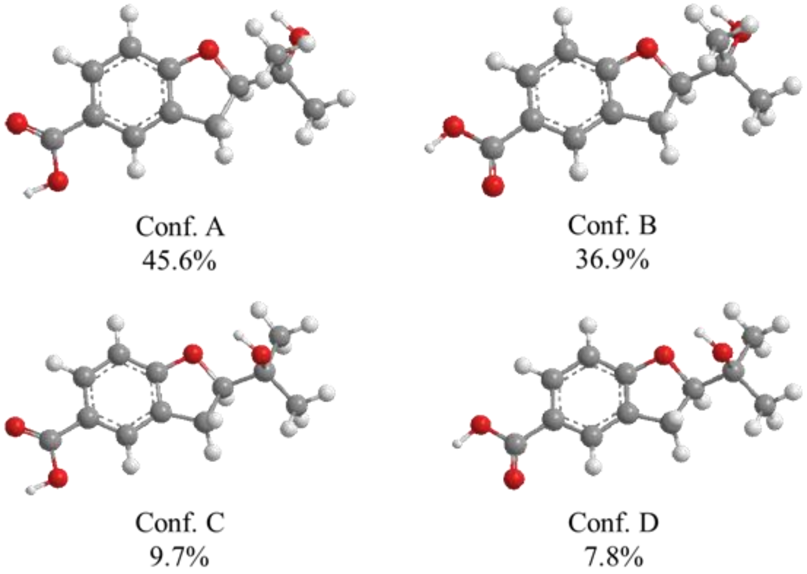

Fig. S28 DFT optimized conformers and populations of compound **4** (*R*) above 2% population.

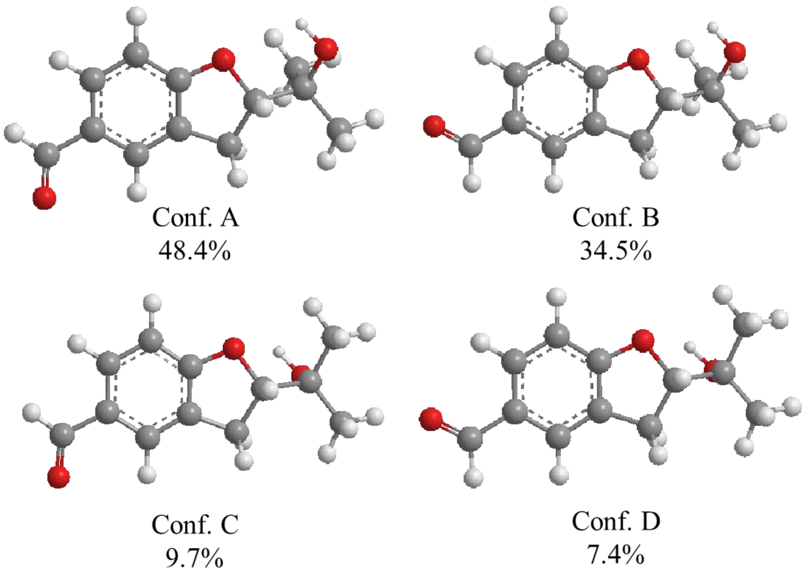

Fig. S29 DFT optimized conformers and populations of compound **5** (*S*)above 2% population.

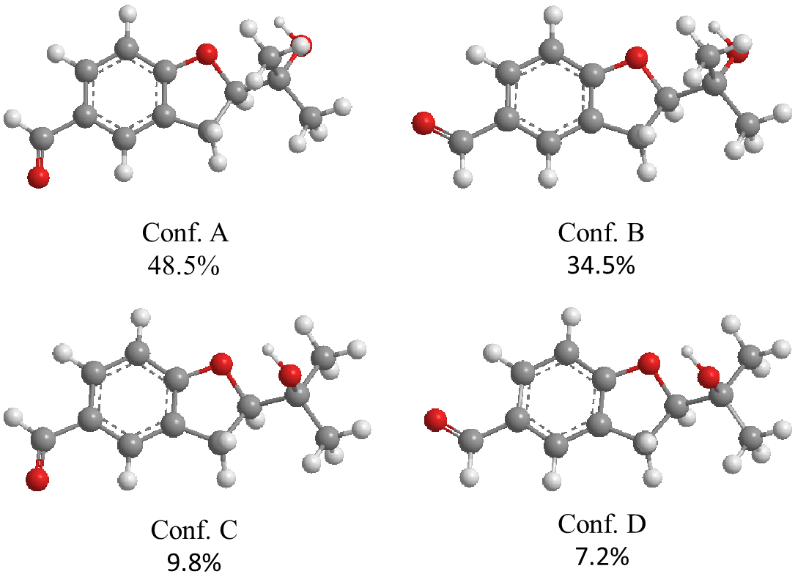

Fig. S30 DFT optimized conformers and populations of compound **5** (*R*) above 2% population.
